# Quantitative phosphoproteomic profiling of CCL5/CCR5 signaling cascade in melanoma cells

**DOI:** 10.3389/fonc.2026.1852022

**Published:** 2026-06-22

**Authors:** Lifen Xie, Tianhui Zhu, An He, Qin Wu, Jinfeng Zeng, Liqin Huang, Ling Zhang, Jie Liu

**Affiliations:** 1 Department of Oncology, The Second Clinical Medical College, Jinan University (Shenzhen People’s Hospital), Shenzhen, China; 2 The First Affiliated Hospital, Jinan University, Guangzhou, China; 3 Department of Chemistry, College of Science, Southern University of Science and Technology, Shenzhen, China; 4 Shenzhen Eye Hospital, Shenzhen Eye Medical Center, Southern Medical University Shenzhen, Guangdong, China; 5 Department of Transfusion Medicine, School of Laboratory Medicine and Biotechnology, Southern Medical University, Guangzhou, China; 6 Shenzhen Blood Center, Shenzhen, China; 7 Immunogenetics Laboratory, Shenzhen Blood Center, Shenzhen, China

**Keywords:** CCL5, CCR5, cell cycle, melanoma, phosphoproteomics, temporal dynamics

## Abstract

**Background:**

The CCL5/CCR5 axis plays a pivotal role in tumor progression and metastasis. We previously reported CCR5 promoted melanoma EMT and metastasis by upregulating TGFβ1 expression via the PI3K/AKT/GSK3β pathway. However, the full spectrum of downstream events triggered by CCR5 activation remains poorly understood.

**Methods:**

Here we employed quantitative phosphoproteomics to profile dynamic phosphorylation events in B16/F10 cells following CCL5 stimulation at three time points (5, 10 and 30 min). Moreover, to specifically dissect CCR5-mediated phosphorylation events distinct from other CCL5 receptors, we performed comparative phosphoproteomics between CCR5 knockout and wild type cells at the 5-min stimulation timepoint.

**Results:**

Temporal analysis revealed that CCL5 treatment modulated 256, 134, and 83 phosphosites at these respective time points. In total, 393 phosphosites across 315 phosphoproteins exhibited significant regulation. The comparative phosphoproteomics between CCR5 knockout and wild type cells identified 52 phosphoproteins and their temporal dynamics were illustrated. Gene Ontology enrichment analysis revealed that CCL5/CCR5 axis participated in various biological processes, particularly cell cycle regulation. Notably, upon CCL5 stimulation, three cell cycle-associated proteins-CEP131, KHDRBS1, and MAPK6-underwent phosphorylation activation, but this activation was remarkably prevented by CCR5 knockout. Overall. these findings provide a comprehensive phosphorylation resource for dissecting the molecular mechanisms underlying CCL5/CCR5-facilitated tumor progression and identifying potential therapeutic targets.

## 1 Introduction

CCL5 (C-C motif chemokine ligand 5), also known as RANTES (regulated upon activation, normal T cell expressed and secreted), is a CC chemokine that plays a pivotal role in the recruitment and activation of immune cells. While CCL5 can bind to CCR1, CCR3, CCR4 and CCR5, it has the highest affinity to CCR5. CCR5 is a G protein-coupled receptor (GPCR) (1), and is recognized as a co-receptor for HIV-1 entrance, leading to the development of CCR5 antagonists such as Maraviroc (2).

CCL5/CCR5 axis has emerged as a pivotal focus in immunology and oncology, with its significance in cancer becoming increasingly evident in the early 2000s. Elevated expression and activation of CCR5 have been observed across a variety of tumor types. Meanwhile, CCL5 can be secreted by diverse cell populations within the tumor microenvironment, including tumor cells, immune cells, endothelial cells and fibroblasts, and it plays a tumor-promoting role through multiple mechanisms (3). Accumulating preclinical evidence has demonstrated the crucial roles of the CCL5/CCR5 axis in cancer progression, metastasis and immune evasion in melanoma, breast cancer, skin squamous cell carcinoma and colorectal cancer (4–7). Recently, Liu et al. identified the CCL5/CCR5 axis as a key mediator of tumor-stromal crosstalk driving cisplatin resistance in the neuroendocrine prostate cancer (8). These reports have spurred clinical investigations into repurposing maraviroc for cancer therapy.

Upon binding to CCR5, CCL5 activates multiple intracellular signaling pathways, including the mammalian target of rapamycin (mTOR) (9), JAK–STAT (10), MEK/ERK (11), PI3K/AKT and NF-κB pathways (12, 13). These pathways regulate diverse cellular processes, such as proliferation, survival, migration, and invasion. Previously, we demonstrated that CCR5 was highly expressed in metastatic melanoma cells and facilitated epithelial-mesenchymal transition (EMT) and metastasis by enhancing the transcription of TGFβ1 through the PI3K/AKT/GSK3β pathway (14). However, the full spectrum of downstream events triggered by CCR5 activation remains poorly understood. Moreover, the molecular mechanisms governing the transcriptional regulation of TGFβ1, as well as other signaling pathways beyond the PI3K-AKT axis, warrant further investigation.

Recent advances in mass spectrometry (MS)-based phosphoproteomics have revolutionized the study of dynamic protein signaling, enabling precise characterization and quantification of phosphorylation changes in cells (15, 16). Phosphoproteomics analysis has been extensively employed to unravel the signaling network downstream of CXCL12/CXCR4 in various disease (17–19), but similar study on CCR5 remains relatively scarce. Despite some degree of overlaps in pathway activation, different chemokines will exhibit distinct signaling events. Factors such as cell type, receptor expression level, G protein availability and disease state also influence the signaling response. Thus, in order to obtain a comprehensive understanding of the downstream signaling cascade triggered by CCR5 activation, we employed a quantitative phosphoproteomic approach to profile the phosphorylation events triggered by CCL5 stimulation in the B16/F10 melanoma cell line at different time points, capturing the temporal dynamics of these modifications. Furthermore, we generated CCR5 knockout B16/F10 cells and performed comparative phosphoproteomic analysis between CCR5 knockout and wild type cells under the same 5 min-CCL5 stimulation condition, to identify phosphoproteins dependent on CCR5. Then these proteins’ temporal phosphorylation dynamics and biological function were illustrated. Notably, three cell cycle-associated proteins-CEP131, KHDRBS1 and MAPK6-were phosphorylated following CCL5 stimulation, but this activation was remarkably prevented by CCR5 knockout.

The phosphoproteomics dataset we believe will provide a valuable resource for dissecting the molecular mechanisms underlying melanoma progression and metastasis facilitated by CCL5/CCR5 axis, and may aid in the identification of potential therapeutic targets for the treatment of melanoma patients.

## 2 Materials and methods

### 2.1 Cell culture, reagents and antibodies

Murine melanoma cells B16/F10 were obtained from the American Type Culture Collection (Manassas, VA, USA) and maintained in DMEM (Corning, 10-014-CVR) supplemented with 10% FBS (PAN, P30-3302) and 1% antibiotic. Recombinant RANTES (CCL5) was obtained from Peprotech (Rocky Hill, CT, USA). The β-actin, AKT, and p-AKT antibodies were all purchased from Cell Signaling Technology (CST, USA) and the catalogs were β-actin (8457), AKT (9272), Phospho-Akt (Ser473) (9272).

### 2.2 Construction of the CCR5 knockout cell line

The CCR5 knockout B16/F10 cell line was established using the CRISPR/Cas9 technology, as detailed in the **Supplementary Materials and Methods**. To assess the knockout efficiency, CCR5 expression was evaluated using flow cytometry with the antibody CD195 (CCR5) Monoclonal Antibody (HM-CCR5 (7A4)), PE, eBioscience™ (Catalog: 12-1957-41).

### 2.3 Cell treatment and preparation of cell lysates

For CCL5-stimulated temporal phosphorylation analysis, B16/F10 cells were serum-starved for 18 h when cell confluence reached 80%. Then cells were stimulated with 200 ng/mL CCL5 for different times (5, 15 and 30 min) with one group remaining no stimulation (0 min). For comparative phosphoproteomics, wild type (WT) and CCR5 knockout (CCR5_KO) cells were serum-starved for 18 h and then stimulated with 200 ng/mL CCL5 for 5 min. Three biological replicates were employed in every experimental group to ensure the reliability and reproducibility of the experimental results. Cell lysates were prepared as described in the **Supplementary Materials and Methods**. Briefly, cells were processed in lysis buffer with protease/phosphatase inhibitors followed by ultrasonic lysis. After centrifugation, the supernatants were collected and protein concentrations were examined.

### 2.4 Sample preparation for phosphoproteomic experiment

Proteins were precipitated from cell lysates using the methanol precipitation method and redissolved in 8M urea. Following reduction and alkylation, proteins were digested with trypsin for 14–18 hours. The resulting peptides were desalted using Sep-Pak Vac tC18 1cc/50mg solid phase extraction (SPE) cartridges (Waters, Milford, Massachusetts, USA). Phosphorylated peptides were enriched via Ti^4+^-immobilized-metal affinity chromatography (Ti-IMAC). After a second desalting step, peptides were redissolved in a solution containing 4% formic acid and 5% acetonitrile prior to mass spectrum analysis. Detailed protocols were provided in the **Supplementary Materials and Methods**.

### 2.5 Quantitative phosphoproteomics analysis

For CCL5-stimulated temporal phosphorylation analysis, the phosphorylated peptides were analyzed with Q Exactive HF-X mass spectrometer equipped with Easy-nanoLC (Thermo Fisher Scientific). For comparative analysis between WT and CCR5_KO cells, the phosphorylated peptides were subjected to the timsTOF Pro mass spectrometry. Detailed protocols and parameters were provided in the **Supplementary Materials and Methods**.

### 2.6 Data processing and statistical analysis

Raw mass spectrometry data acquired from the Q Exactive HF-X were processed using MaxQuant (v1.5.5.1), while data-independent acquisition (DIA) data from the timsTOF Pro were analyzed with Spectronaut (v.18). For phosphopeptide identification, oxidation (M), deamidation (NQ) and phosphorylation (S/T/Y) were included as variable modifications. Carbamidomethylation (C) was specified as a fixed modification. Trypsin/P was specified as the cleavage enzyme, allowing up to two missing cleavages. Peptide and protein identifications were filtered at a false discovery rate (FDR) of 1%.

Statistical comparisons between different groups were performed using Perseus (Version 1.5.5.3). Student’s t-test was applied to identify significantly regulated phosphosites with the threshold of FDR as 0.05 and S0 as 0.5. In order to chart dynamic change of phosphopeptide intensity, intensities were normalized and the relative abundance to the maximum value among all time points of every phosphopeptide was calculated. Then the dynamic changes in phosphopeptide abundance were visualized in heatmaps through R packages (Version 3.6.1). Gene Ontology (GO) enrichment analysis was conducted using the clusterProfiler R package (v4.2.2) and Reactome pathways enrichment was performed via the String database. Detailed methodological parameters were provided in the **Supplementary Materials and Methods**.

## 3 Results

### 3.1 Experimental design and quality control evaluation

**Figure 1:**
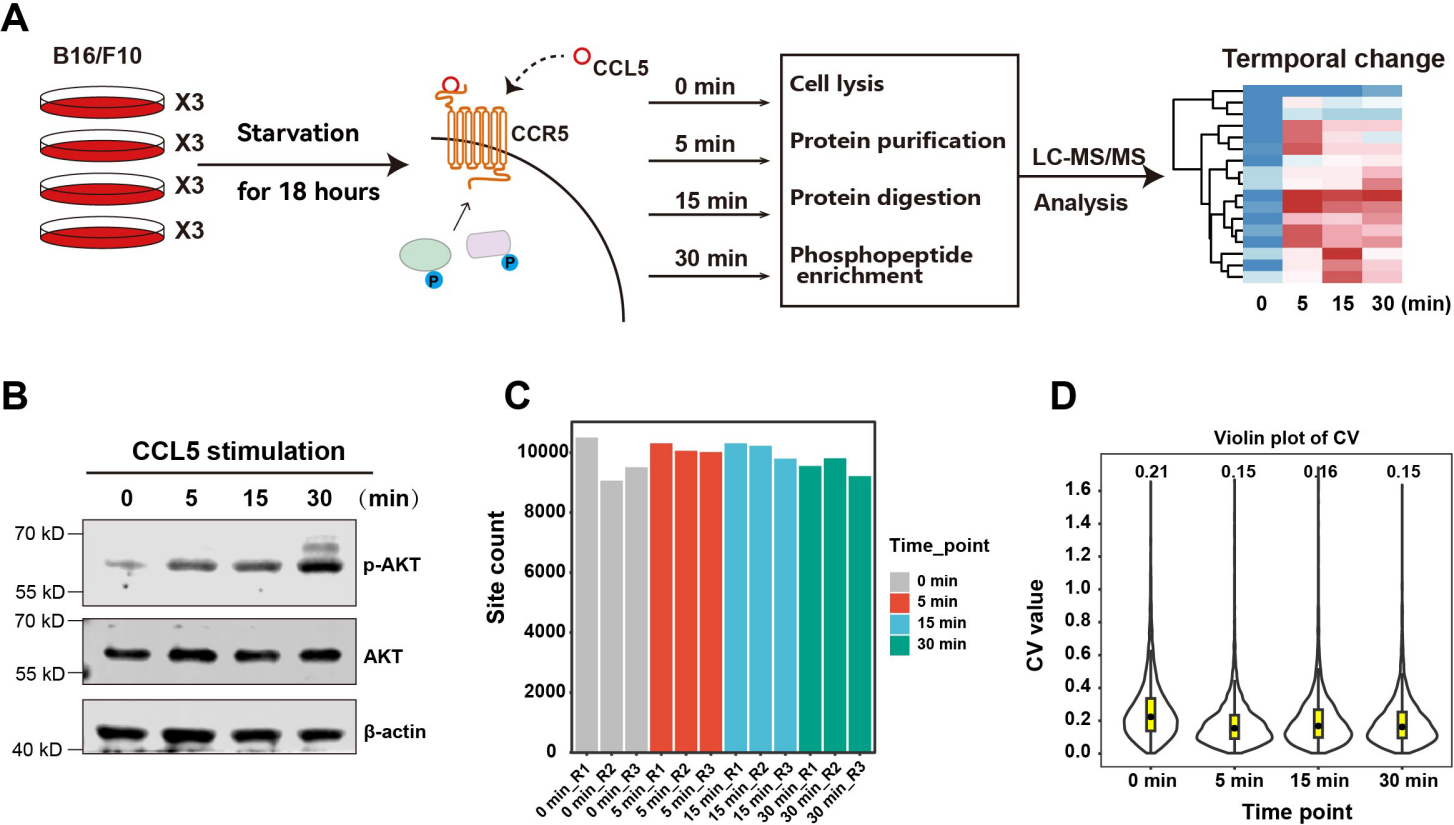
Experimental design and quality control evaluation. **(A)** Workflow schematic of exploring CCL5/CCR5 regulated dynamic phosphoproteome in melanoma cells. **(B)** The protein levels of phospho-AKT (Ser473), AKT and β-actin were detected through western blot assay following CCL5 stimulation for 5, 15 and 30 min in B16/F10 cells. **(C, D)** The reproducibility evaluation of phosphoproteomic experiments. The identified phosphosite counts **(C)** and the coefficient of variation (CV) distribution of phosphopeptide intensities **(D)** among three biologically independent replicates were calculated.

The workflow was illustrated in **Figure 1A**. Briefly, B16/F10 cells with high expression of CCR5 (14) were untreated (0 min) or stimulated with its high-affinity ligand CCL5 for 5, 15 and 30 min after serum starvation for 18 h. After cell lysis, protein purification, tryptic digestion and Ti-IMAC enrichment, phosphopeptides were analyzed using liquid chromatography-tandem mass spectrometry (LC-MS/MS). With MaxQuant search and Perseus analysis, CCL5 stimulation modulated phosphosites were identified and their temporal changes were charted after quantitative analysis.

The phosphorylated AKT at Ser 473 has been previously reported to be activated by CCR5 activation (12, 14, 20) and was detected here to estimate CCL5 stimulation efficiency. As shown in **Figure 1B**, p-AKT was significantly enhanced after CCL5 stimulation and exhibited a time-dependent manner, which validated the success of CCR5 activation. Furthermore, to evaluate the reproducibility of proteomics experiments, the identified phosphosite counts (**Figure 1C**), coefficient of variation (CV) distributions (**Figure 1D**) and Pearson correlations (**Supplementary Figure 1**) of phosphopeptide intensities among three biologically independent replicates were calculated. These data suggested the reliability of the phosphoproteomic results, which laid the foundation for the further analysis.

### 3.2 Temporal phosphorylation dynamics of CCL5-regulated proteins in melanoma cells

**Figure 2:**
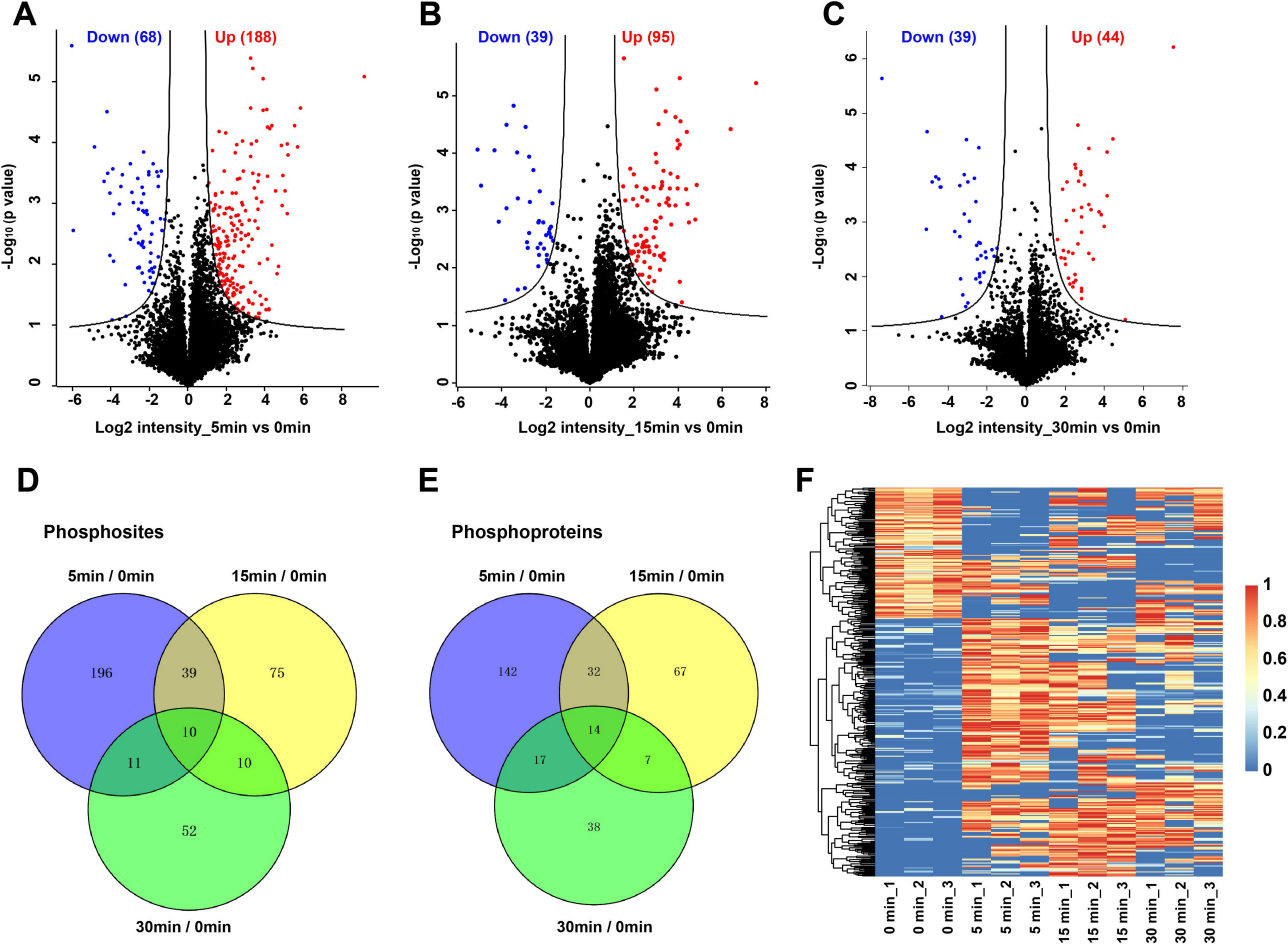
Temporal phosphorylation dynamics of CCL5-regulated proteins in melanoma cells. **(A–C)** Volcano plots showing modulated phosphosites by CCL5 stimulation at different time points: 5 min **(A)**, 15 min **(B)** and 30 min **(C)**) in melanoma cells (FDR = 0.05 and S_0_ = 0.5). **(D, E)** Venn diagrams showing the counts of unique and shared phosphosites **(D)** and phosphoproteins **(E)** regulated by CCL5 stimulation for 5, 15 and 30 minutes. **(F)** Heatmap visualization of temporal intensity changes across 393 phosphosites with significant regulation (p < 0.05) following CCL5 stimulation.

A total of 17060 phosphosites in 4057 phosphoproteins were identified (**Supplementary Table 1**), most of which have not been previously implicated in CCL5/CCR5 signaling. To determine specific phosphosites modulated by CCL5 stimulation, log_2_-transformed intensities at 5, 15 and 30 min were compared with those in the control group (0 min) with the Perseus software. Significantly changed phosphosites with the threshold of FDR = 0.05 and S_0_ = 0.5 were selected and highlighted in red (up-regulated) or blue (down-regulated) (**Figures 2A–C**). CCL5 stimulation modulated 256 phosphosites in 203 proteins at 5 min, which changed to 134 phosphosites across 120 proteins at 15 min and 83 phosphosites across 76 proteins at 30 min (**Supplementary Table 2**). The up-regulated phosphosites were more abundant than down-regulated ones at three time points, with peak values reaching at the short time point (5 min). The distinct and shared phosphosites and phosphoproteins regulated by CCL5 stimulation at 5, 15 and 30 min were quantified and displayed in Venn diagrams, as shown in **Figures 2D, E**.

Collectively, a total of 393 phosphosites across 315 phosphoproteins were regulated by CCL5 stimulation. To investigate the dynamic change of these phosphosites across the stimulation time course, intensities were normalized and the relative abundance to the maximum value among all time points of every phosphopeptide was calculated (**Supplementary Table 3**), exhibited in the heatmap diagram (**Figure 2F**).

### 3.3 Biological function analysis of modulated phosphoproteins by CCL5 stimulation

**Figure 3:**
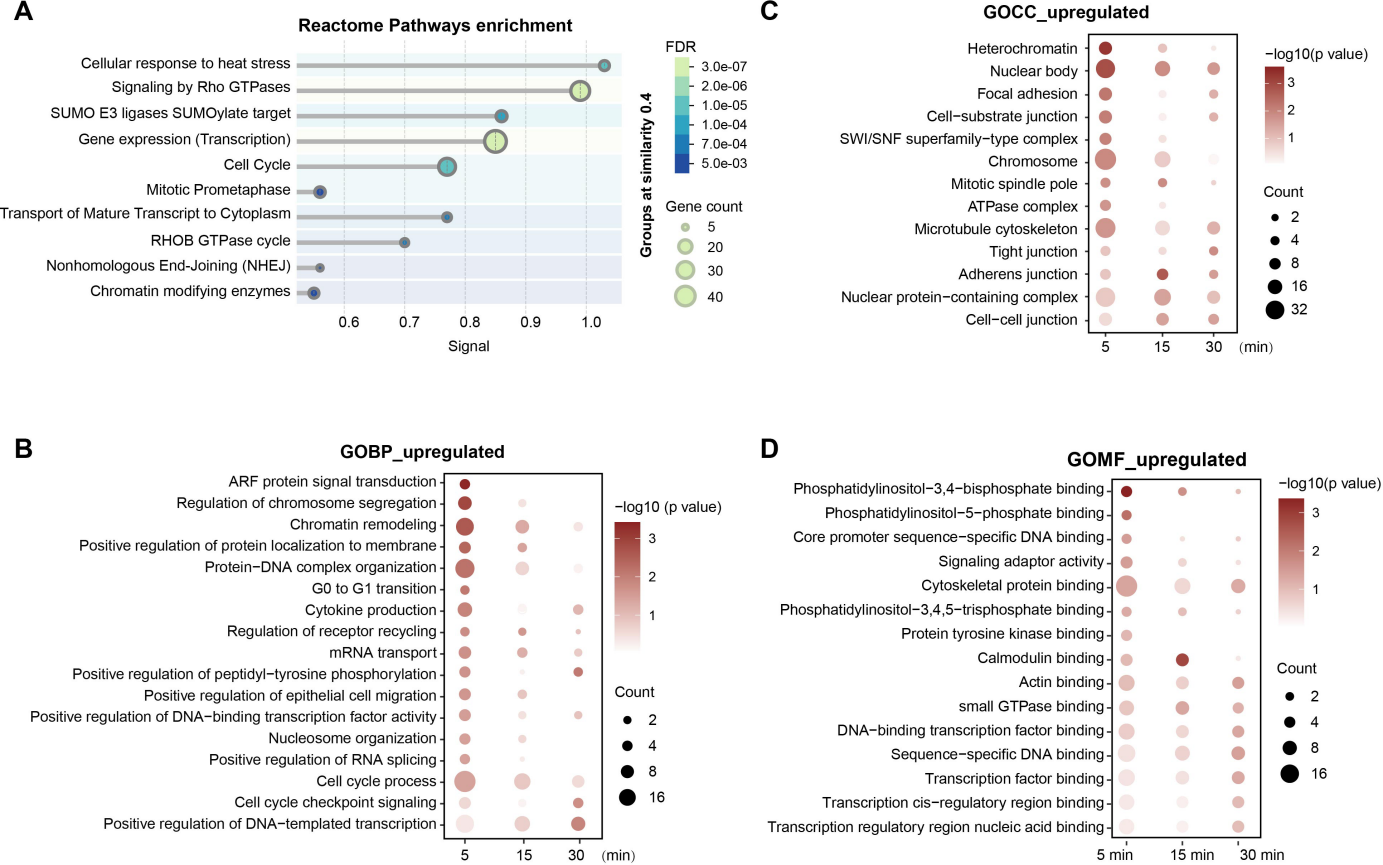
Biological function analysis of modulated phosphoproteins by CCL5 stimulation. **(A)** Reactome pathways enrichment analysis of regulated phosphoproteins induced by CCL5 stimulation. Groups were categorized with similarity of 0.4 using String database. **(B–D)** Comprehensive Gene Ontology (GO) functional profiling of the dynamic CCL5-responsive phosphoproteome, stratified into biological processes **(B)**, cellular components **(C)**, and molecular functions **(D)**. All significantly enriched terms (FDR < 0.05) were displayed with -log_10 (_p value_)_ metrics and gene counts.

**Figure 4:**
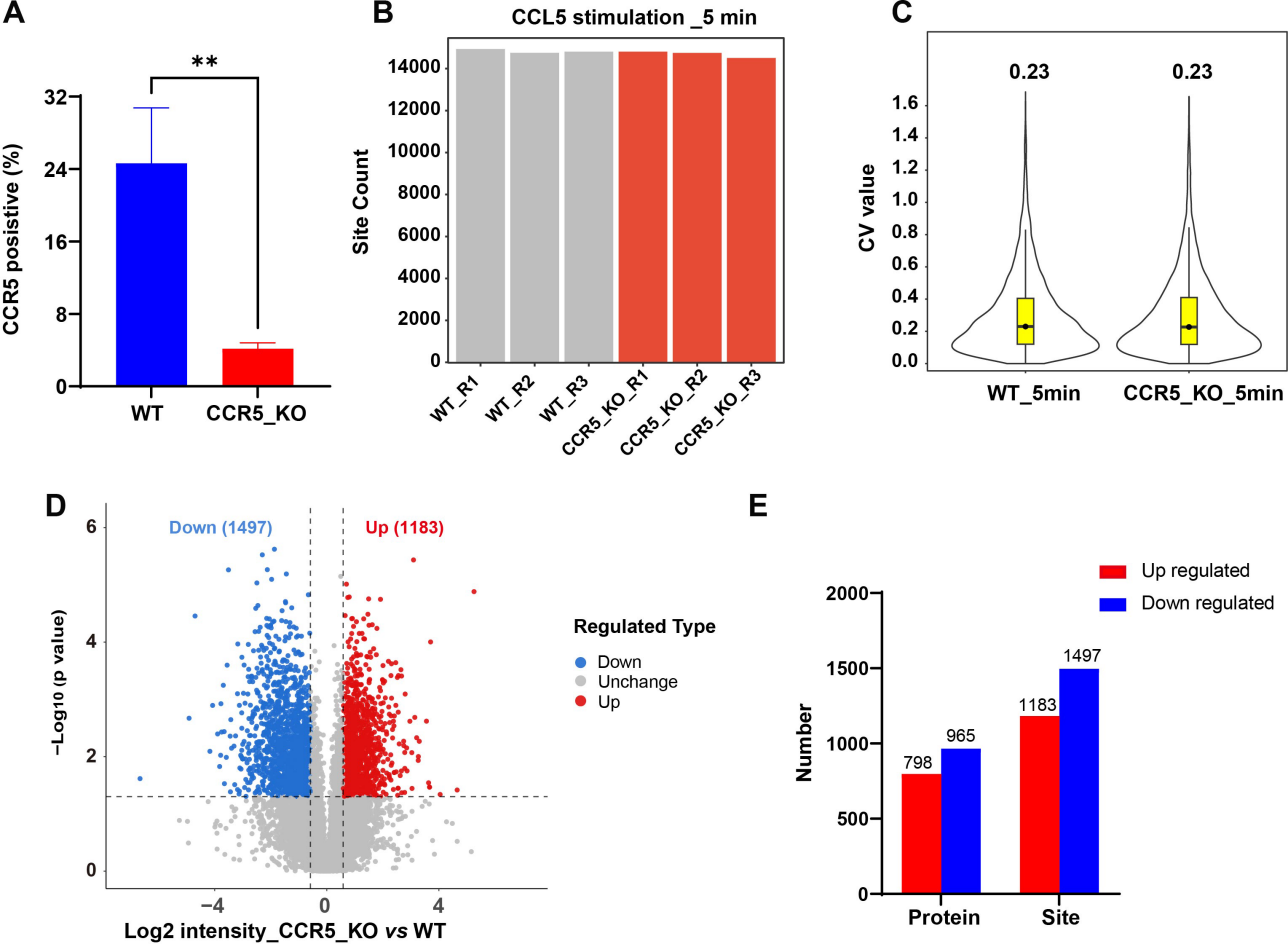
Comparative phosphoproteomics between CCR5 knockout and wild type cells. **(A)** Surface expression of CCR5 protein was quantified using flow cytometry, with the y-axis indicating the proportion of CCR5-positive cells. Statistical comparisons between WT and KO groups were performed via unpaired Student’s t-test. **p < 0.01. **(B, C)** The reproducibility of the phosphoproteomic experiments was evaluated. Specifically, the number of identified phosphosites **(B)** and the coefficient of variation (CV) distribution of phosphopeptide intensities **(C)** were calculated across three biologically independent replicates. **(D)** Volcano plots showing modulated phosphosites by CCR5 knockout at the 5-minute timepoint post CCL5 stimulation (FDR = 0.05 and S_0_ = 0.5). **(E)** The quantity of significantly regulated phosphoproteins and phosphosites identified in a comparative analysis between WT and CCR5_KO cells. The blue represented up-regulated ones and the red represented down-regulated ones resulting from CCR5 knockout.

CCL5 primarily interacts with CCR5, and the relative expression levels of other CCL5 receptors-CCR1, CCR3 and CCR4-in B16/F10 cells were markedly lower than that of CCR5 (**Supplementary Figure 2**). Therefore, the effects elicited by CCL5 stimulation can be predominantly attributed to the activation of CCR5. The classified proteins with regulated phosphorylation in response to CCL5 stimulation were subjected to Reactome Pathway enrichment analysis using the String database. As illustrated in **Figure 3A**, these proteins were predominantly implicated in heat stress response, Rho GTPase signaling, gene expression, cell cycle progression, chromatin modification. We further narrowed our focus to up-regulated phosphoproteins and performed Gene Ontology (GO) analysis on them. The results depicted in **Figure 4B** revealed their involvement in ARF protein signal transduction, chromatin remodeling, cell cycle, receptor recycling, cell migration, peptidyl-tyrosine phosphorylation, and transcription regulation. Consistently, GO Cellular Component (GOCC) analysis indicated that these proteins were predominantly located in chromatin remodeling complexes such as nuclear body, heterochromatin, and SWI/SNF superfamily-type complex; in structures associated with cell adhesion and migration such as focal adhesion, microtubule cytoskeleton and adherens junction; as well as ATPase complexes and mitotic spindle poles (**Figure 4C**). Furthermore, GO Molecular Function (GOMF) analysis (**Figure 4D**) highlighted the involvement of these proteins in PI3K signaling, signaling adapter activity, cytoskeleton regulation, protein tyrosine kinase binding, calmodulin binding, small GTPase binding and transcription regulation.

These findings provide valuable insights into the complex regulatory network governed by CCL5 stimulation and offer a foundation for future investigations into its therapeutic potential.

### 3.4 Comparative phosphoproteomics between CCR5 knockout and wild type cells

To exclude the minimal contribution of other CCL5 receptors despite their notable lower expression levels, we established a CCR5 knockout (CCR5_KO) B16/F10 cell line using CRISPR/Cas9 technology. The knockout efficiency was assessed via flow cytometry, as depicted in **Figure 4A** and **Supplementary Figure 3**. Next, the comparative phosphoproteomic experiment was performed between the wild type (WT) and CCR5_ KO cells, both stimulated with CCL5 for 5 min. The consistency across three biologically independent replicates was evaluated by calculating the identified phosphosites count (**Figure 4B**) and CV distribution of phosphopeptide intensities (**Figure 4C**). To identify phosphoproteins specifically regulated by CCR5, significantly changed phosphosites in CCR5_KO cells relative to the WT cells were selected and visualized in volcano plots, as shown in **Figure 4D**. In total, 1183 phosphosites across 798 proteins were significantly up-regulated following CCR5 knockout, whereas 1497 phosphosites in 965 proteins were notably down-regulated (**Supplementary Table 4**).

### 3.5 Protein phosphorylation regulated by the CCL5/CCR5 axis and associated biological functional analysis

**Figure 5:**
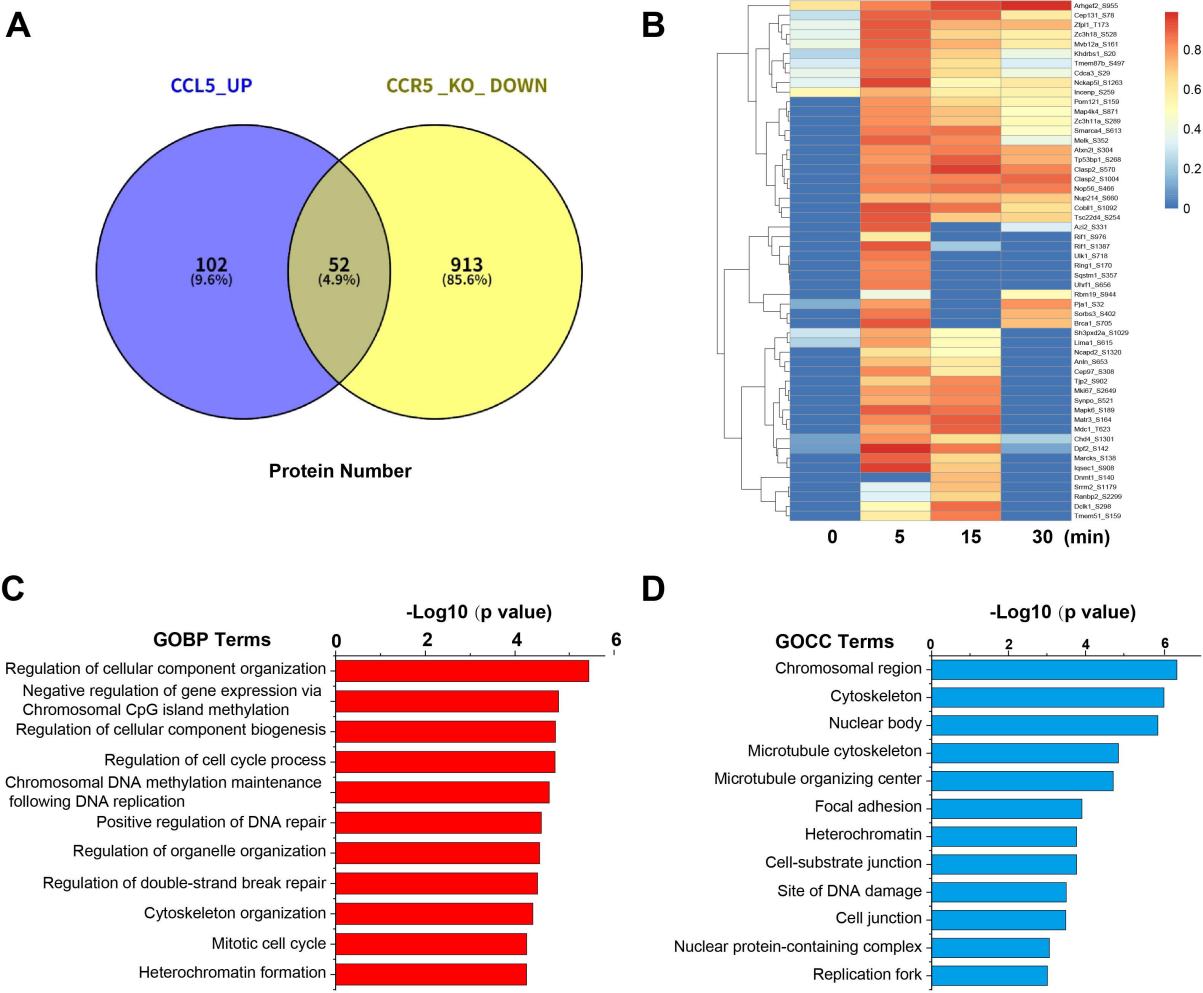
Protein phosphorylation regulated by the CCL5/CCR5 axis and associated biological functional analysis. **(A)** Venn diagrams illustrating the number of phosphoproteins uniquely upregulated by 5-minute CCL5 stimulation (purple), uniquely downregulated by CCR5 knockout (yellow), and shared between both conditions (overlap). **(B)** Heatmap visualization of temporal dynamics in phosphorylation intensities for significantly modulated phosphosites by CCL5/CCR5 axis. **(C, D)** Gene Ontology (GO) biological process **(C)** and cellular component **(D)** enrichment analysis of 52 phosphoproteins regulated by the CCL5/CCR5 axis. Every enriched GO term was displayed with the corresponding -log_10_ (p value).

To identify phosphoproteins specifically upregulated by the CCL5/CCR5 axis, we defined the intersection of two sets: (i) phosphoproteins with increased phosphorylation levels in WT cells following 5 min of CCL5 stimulation relative to unstimulated WT cells; and (ii) phosphoproteins with decreased phosphorylation levels in CCR5_KO cells compared to WT cells under the same 5 min-CCL5 stimulation condition. This intersection yielded 52 phosphoproteins that were both CCL5-responsive and CCR5-dependent (**Figure 5A**, **Supplementary Table 5**). Their temporal dynamics were visualized with heatmap diagram as shown in **Figure 5B**. Furthermore, these proteins were subjected to GO enrichment analysis to uncover their biological functions. GOBP enrichment analysis illustrated that these proteins were involved in cellular component organization, gene expression, cell cycle regulation, DNA repair and cytoskeleton organization (**Figure 5C**). Meanwhile, GOCC enrichment analysis demonstrated that they predominantly localized in the chromosomal region, cytoskeleton, focal adhesion, DNA damage sites, and replication forks (**Figure 5D**).

Collectively, these findings indicate that CCL5/CCR5 axis may play a critical role in the regulation of gene expression, DNA repair, cell adhesion, cytoskeletal rearrangement and especially cell cycle process.

### 3.6 The CCL5/CCR5 axis enhanced the phosphorylation of proteins involved in cell cycle process

**Figure 6:**
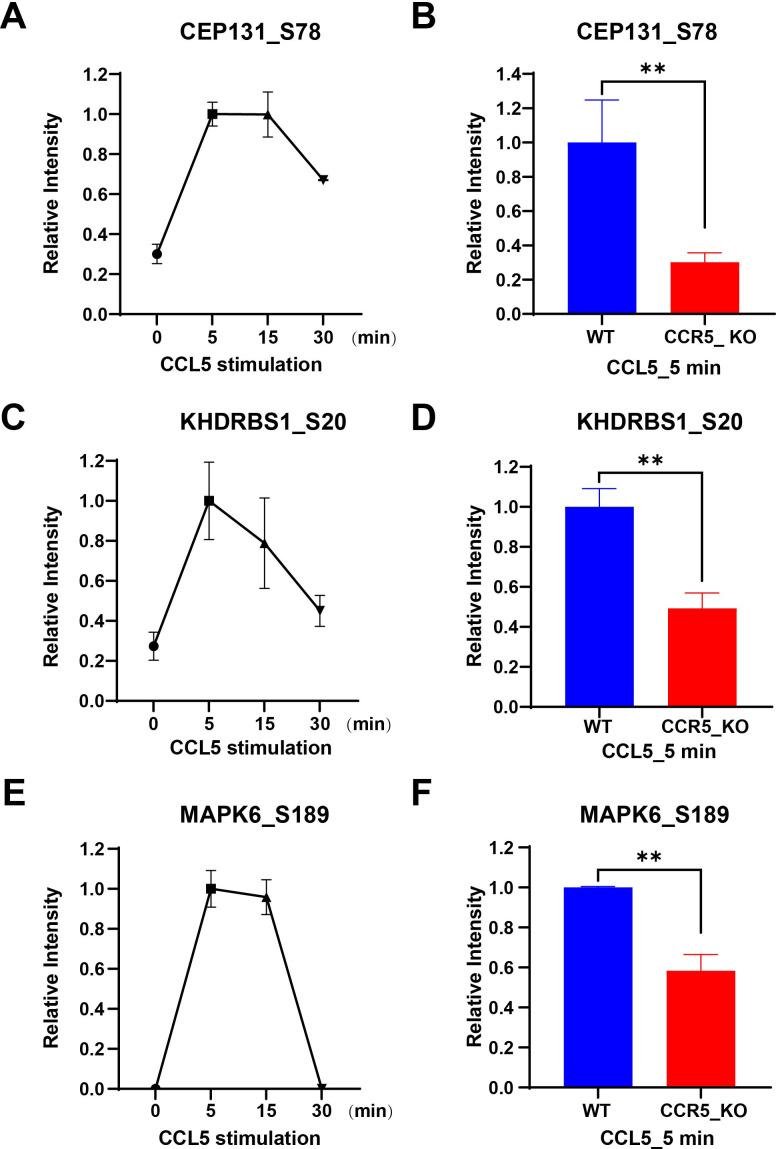
The CCL5/CCR5 axis enhanced the phosphorylation of proteins involved in cell cycle process. **(A)** Temporal phosphorylation dynamics of CEP131 at serine 78 (CEP131_S78) following CCL5 stimulation at 0, 5, 15 and 30 min. **(B)** Comparative analysis of CEP131_S78 phosphorylation intensity at the 5-minute CCL5 stimulation timepoint between WT and CCR5_KO cells. **(C)** Temporal phosphorylation dynamics of KHDRBS1 at serine 20 (KHDRBS1_S20) following CCL5 stimulation at 0, 5, 15 and 30 min. **(D)** Comparative analysis of KHDRBS1_S20 phosphorylation intensity at the 5-minute CCL5 stimulation timepoint between WT and CCR5_KO cells. **(E)** Temporal phosphorylation dynamics of MAPK6 at serine 189 (MAPK6_S189) following CCL5 stimulation at 0, 5, 15 and 30 min. **(F)** Comparative analysis of MAPK6_S189 phosphorylation intensity at the 5-minute CCL5 stimulation time point between WT and CCR5_KO cells. Data represent mean ± SD (n=3), with statistical significance assessed by Student’s t-test **p < 0.01.

Next, we further explored the regulation of CCL5/CCR5 axis on cell cycle-related phosphoproteins. Cep131 encodes the protein Centrosomal Protein 131 (CEP131), which plays critical roles in optimal cell proliferation and cell cycle progression through facilitating centriole duplication (21, 22). In our study, upon CCL5 stimulation, the phosphorylation level of CEP131 at Ser78 rapidly increased within 5 minutes and sustained a high level until 15 minutes before declining at 30 minutes (**Figure 6A**). However, the phosphorylation of CEP131_S78 was remarkably prevented by CCR5 knockout (**Figure 6B**).

Khdrbs1 encodes the protein KHDRBS1 (KH Domain Containing, RNA Binding Signal Transduction Associated 1), also known as Sam68 (Src-associated in mitosis 68 kDa protein), which acts as an adapter protein in signal transduction cascades by binding to proteins containing SH2 and SH3 domains, ultimately regulating cell cycle progression (23, 24). Our findings indicated that the phosphorylation of KHDRBS1 at Ser20 was promptly induced by CCL5 stimulation, peaking at 5 minutes, and then gradually diminished with prolonged stimulation (**Figure 6C**). Furthermore, this phosphorylation increase was CCR5-dependent, as CCR5 deletion markedly suppressed the phosphorylation elevation (**Figure 6D**).

Mapk6 encodes the protein mitogen-activated protein kinase 6 (MAPK6), also known as extracellular signal-regulated kinase 3 (ERK3), which is an atypical member of the MAPKs and mainly regulated by protein stability, subcellular localization, and specific phosphorylation-dependent interactions (25). MAPK6 specially interacts with MAPK-activated protein kinase 5 (MK5), which leads to the phosphorylation of both MAPK6 and MK5 (26), ultimately resulting in further physiological functions including cell cycle regulation (27). We observed that the phosphorylation level of MAPK6 at Ser189 rapidly peaked at approximately 5 minutes, remained relatively high at 15 minutes with a slight decrease, and nearly returned to baseline by 30 minutes (**Figure 6E**). Nevertheless, CCR5 deletion impeded the increase in phosphorylation (**Figure 6F**).

The above results revealed that the CCL5/CCR5 axis markedly promoted the phosphorylation of proteins involved in cell cycle process. This finding suggests that CCL5/CCR5 may play a pivotal role in cell cycle regulation, which was consistent with previous reports (28–30). Nevertheless, further functional studies are necessary to confirm the specific roles of CEP131, KHDRBS1 and MAPK6 phosphorylation in CCR5-mediated cell cycle progression.

### 3.7 Kinase prediction and sequence motif analysis of the three phosphorylation sites

To comprehensively investigate the upstream regulatory mechanism of the three identified phosphorylation sites, we first queried the PhosphoSitePlus database for experimentally validated upstream kinases of each phosphosite. For CEP131_S78, MAPK-activated protein kinase 2 (MK2) (22) and Polo-like kinase 4 (PLK4) (31) were confirmed as validated regulators. For MAPK6_S189, autophosphorylation by MAPK6 itself (32) and phosphorylation by p21-activated kinase 2 (PAK2) (33) have been documented. No experimentally verified upstream kinase was reported for KHDRBS1_S20.

To complement these experimental data, kinase family prediction was performed using GPS 6.0 with a high-confidence threshold, combined with motif analysis of the local sequence context. The results were summarized in **Table 1**. For CEP131_S78, the top-ranked kinase families were CAMK (Score = 0.9990) and TKL (Score = 0.9988), which aligned with the validated kinases MK2 (a CAMK family member) and PLK4 (an AGC family kinase). The sequence surrounding CEP131_S78 contains a canonical basophilic R-X-X-S motif, consistent with the substrate preference of CAMK family kinases such as MK2. For MAPK6_S189, both CAMK (Score = 0.1588) and AGC (Score = 0.0905) families exceeded the high-confidence cutoff. The peptide sequence SHKGHGLS centered at Ser189 harbors a basophilic H-X-X-S motif, which is compatible with the substrate preferences of both CAMK and AGC kinases. For KHDRBS1_S20, for which no experimentally reported regulator exists, GPS 6.0 identified the AGC family as the highest-confidence candidate (Score = 0.9732). The local sequence SSGRSCSKDPSGA contains a conserved R-X-X-S motif, matching the preferred substrate specificity of AGC kinases such as SRPK and PKC.

**Table 1:** Kinase family prediction for the phosphosites CEP131_S78, KHDRBS1_S20 and MAPK6_S189 using GPS 6.0.

Phosphosite	Sequence	Predicted kinase family	Score	Cutoff (high confidence)
CEP131_S78	NLRRSNSTTQVNQ	CAMK	0.9990	0.04
		TKL	0.9988	0.9215
		PKL	0.9074	0.3137
		AGC	0.7184	0.0478
		CK1	0.3742	0.0996
		RGC	0.2915	0.097
MAPK6_S189	SHKGHLSEGLVTK	CAMK	0.1588	0.04
		AGC	0.0905	0.0478
KHDRBS1_S20	SSGRSCSKDPSGA	AGC	0.9732	0.0478
		PKL	0.5363	0.3137
		RGC	0.1113	0.0970

**Figure 7:**
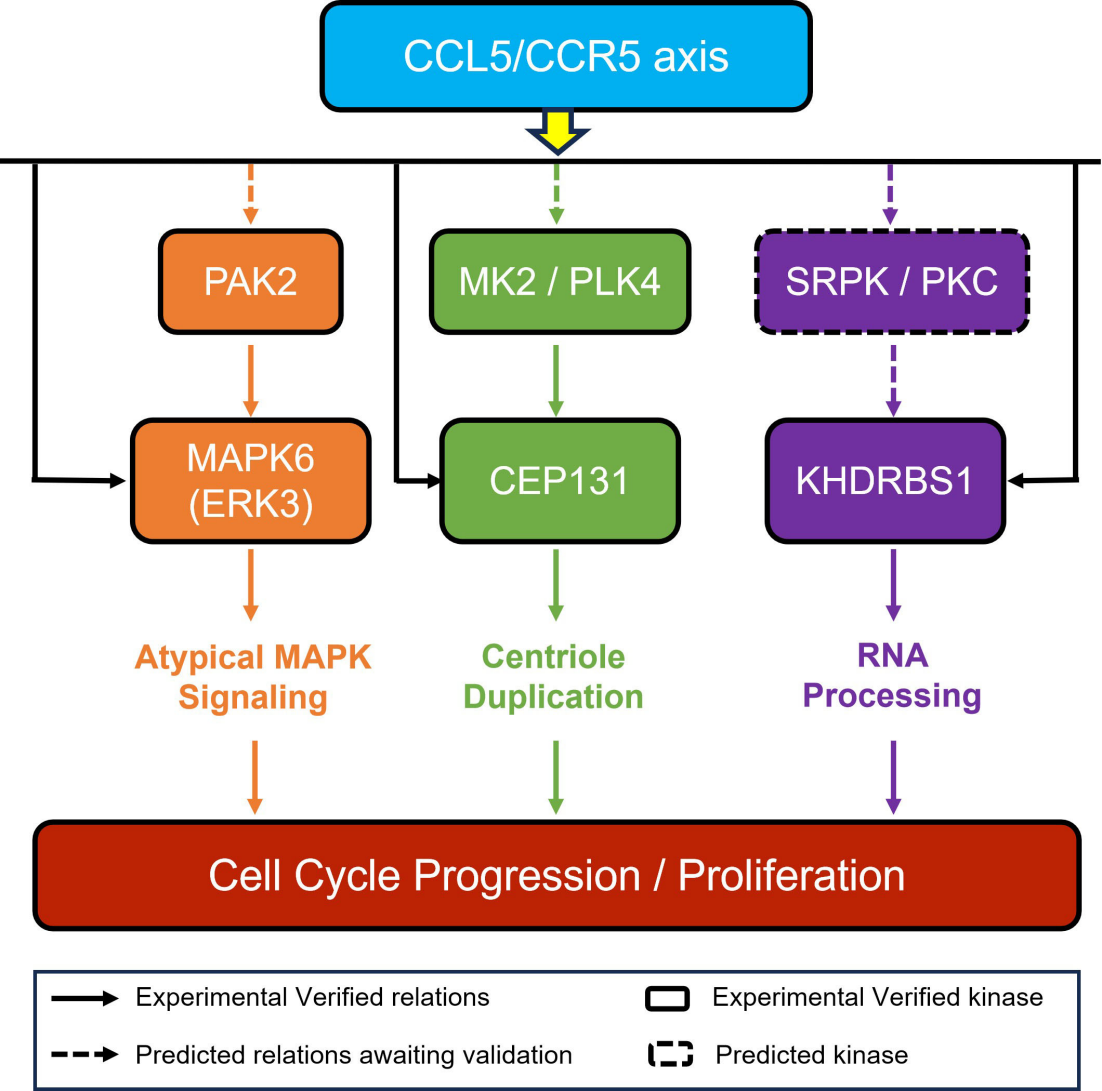
A working model of CCR5-mediated multi-branch non-canonical signaling networks regulating cell cycle progression. It was predicted that CCR5 activated three functionally distinct non-canonical modules: a PAK2-MAPK6 axis (atypical MAPK signaling), an MK2/PLK4-CEP131 module (centriole duplication), and an SRPK/PKC-KHDRBS1 axis (RNA processing), collectively promoting cell cycle progression and proliferation. Predicted kinase–substrate relationships was presented as dashed arrows awaiting experimental validation.

According to above results, we summarized a working model of CCL5/CCR5-mediated multi-branch signaling networks regulating cell cycle progression. As illustrated in **Figure 7**, three functionally distinct modules were proposed: (1) a PAK2-MAPK6 axis linking CCR5 to non-classical MAPK signaling, (2) an MK2/PLK4-CEP131 module that may impinge on the centriole duplication checkpoint at the G1/S transition; and (3) an AGC kinase (SRPK/PKC)-KHDRBS1 axis coupling receptor activation to cell cycle entry via RNA processing. While these kinase-substrate relationships await experimental validation, our study provides novel cues for expanding the repertoire of CCR5-regulated signaling events.

## 4 Discussion

Chemokines regulate diverse cellular processes, including proliferation, migration, adhesion, and survival by engaging cognate receptors and initiating intracellular signaling cascaded. While phosphoproteomics has been extensively employed to unravel the signaling network downstream of CXCL12/CXCR4 (17–19), the downstream signaling of CCR5 remains less characterized. The present study employed quantitative phosphoproteomics to provide a system-wide view of CCL5/CCR5 signaling in melanoma cells. We quantified 17060 phosphosites across 4057 phosphoproteins, identifying 393 sites on 315 proteins regulated by CCL5 stimulation, most of which were previously unlinked to CCL5 signaling. Furthermore, by comparing CCR5_KO and WT cells under identical 5-minute CCL5 stimulation, we identified 52 phosphoproteins whose phosphorylation level was specifically enhanced by CCL5/CCR5 axis. We characterized the temporal dynamics of these proteins and provided detailed insights into the three-cell cycle-associated proteins: CEP131, KHDRBS1, and MAPK6. This dataset we believe will serve as a valuable phosphoproteomic resource for further investigations into the molecular mechanism underlying CCL5/CCR5 regulated biological process.

Although Vacchini et al. previously mapped CCR5 signaling, they primarily focused on contrasting CCR5 with ACKR2 and delineating differential signaling kinetics (34). Their observation that CCR5 activation induced rapid phosphorylation (peaking at 3 min) aligned with our findings, validating the swift initiation of cascades upon receptor engagement.

While CCL5 can also bind to CCR1, CCR3, and CCR4, it has the highest affinity to CCR5 (1). Moreover, our results showed that the expression level of CCR5 was significantly higher than that of other CCL5 receptors in B16/F10 cells (**Supplementary Figure 2**). Therefore, the effects elicited by CCL5 stimulation can be predominantly attributed to CCR5 activation in B16/F10 cells. Reactome pathway and GO enrichment analyses indicated that CCL5-induced phosphoproteins were involved in heat stress responses, small GTPase signaling, transcription, chromatin modification, cell cycle, receptor recycling, cytoskeleton remodeling and cell migration, among which the majority have been previously reported in CCL5/CCR5 signaling (28, 35, 36). Notably, our finding that the CCL5/CCR5 axis participated in heat stress response corroborated CCR5’s role in acute immune response (37). Additionally, the enrichment of phosphorylated proteins in nuclear bodies and chromatin complexes was consistent with the established role of CCL5/CCR5 in regulating gene expression such as IL-6 (38, 39) and TGFβ1 (14), providing mechanistic insights into CCR5-mediated transcriptional regulation.

To exclude contributions of other CCL5 receptors despite their significantly lower expression, we performed comparative phosphoproteomics on CCR5_KO *vs* WT cells under identical 5-minute CCL5 stimulation (the peak response time). This stringent approach identified 52 phosphoproteins specifically up-regulated by the CCL5/CCR5 axis. Enrichment analysis revealed that these proteins were predominately involved in gene expression, cell cycle regulation, DNA repair, cell adhesion and cytoskeleton rearrangement. Consistently, it was reported that CCR5 antagonist maraviroc prevented metastasis of pancreatic cancer and breast cancer via cell cycle inhibition (28, 30). Tang et al. demonstrated that CCR5 blockade suppressed IL-6/STAT3 signaling and tumor growth in melanoma cells (39). CCR5 enhanced DNA repair to protect tumors from chemotherapeutic agents-induced apoptosis in neuroendocrine prostate cancer and breast cancer (8, 40). All these findings substantiate the reliability of our dataset.

We further focused on the phosphorylation regulation of CCL5/CCR5 axis on cell cycle related proteins. Specifically, the CCL5/CCR5 axis rapidly and dynamically regulated the phosphorylation of CEP131 (Ser78), KHDRBS1 (Ser20), and MAPK6 (Ser189). CEP131 is a centriolar satellite protein essential for centriole duplication and genomic stability, and its phosphorylation by PLK4 at Ser78 was known to facilitate centrosome maturation and mitotic entry (21, 31, 41). KHDRBS1 (Sam68) functions as an SH2/SH3 domain-containing adapter protein that regulates cell cycle progression and proliferation, particularly at the G1/S transition and during mitosis (23, 24, 42). MAPK6 (ERK3), while not a classical MAPK, has been implicated in cell cycle regulation through its interaction with MK5, its C-terminal domain phosphorylation controlled by Cdk1 and Cdc14, and its role in cell proliferation (26, 27, 43). Therefore, the coordinated phosphorylation of these three proteins positions CCR5 as an upstream regulator capable of simultaneously modulating multiple nodes within the cell cycle machinery.

Integrated with kinase prediction, our study uncovered a multi-branch non-canonical signaling network downstream of CCR5 that extends beyond the classical Gαi-MAPK/ERK1/2 cascade. The predicted PAK2-MAPK6 axis, MK2/PLK4-CEP131 module and AGC kinase (SRPK/PKC)-KHDRBS1 axis indicated that CCL5/CCR5 signaling potentially orchestrated cell cycle progression by simultaneously engaging atypical MAPK signaling, centriole homeostasis and RNA metabolism. Although experimental validation of these kinase-substrate relationships and further functional investigation are still required, our study provides a new foundation for exploring the expanded signaling landscape downstream of CCR5 and offers mechanistic insights with potential therapeutic implications for melanoma.

## 5 Conclusions

In summary, our study systematically characterized the temporal dynamics of protein phosphorylation following CCL5 stimulation in melanoma cells and identified three cell cycle-related proteins that specifically depend on CCR5 signaling. This resource not only advances our understanding of CCL5/CCR5 mediated signaling, but also provides a valuable foundation for identifying potential therapeutic targets in melanoma treatment.

## Data Availability

The data that support the findings of this study are available in the article/**Supplementary Material**. The mass spectrometry proteomics data have been deposited to the ProteomeXchange Consortium via the PRIDE partner repository with the dataset identifier PXD066097. Further inquiries can be directed to the corresponding authors.

## Supplementary material

Supplementary Table 1 List of identified phosphosites.

Supplementary Table 2 List of significantly changed phosphosites after CCL5 stimulation at 5,15 and 30 min.

Supplementary Table 3 The relative abundance to the maximum value among 4 timepoints for 393 modulated phosphosites.

Supplementary Table 4 List of modulated phosphosites in CCR5_KO cells compared to WT cells with CCL5 stimulation for 5min.

Supplementary Table 5 List of 52 phosphoproteins up-regulated by CCL5 -CCR5 axis.
