# Supplementary material for: Quantitative phosphoproteomic profiling of CCL5/CCR5 signaling cascade in melanoma cells
Source: Front Oncol. 2026 Jun 22;16:1852022. doi: 10.3389/fonc.2026.1852022 (PMC13333406; doi:10.3389/fonc.2026.1852022)
Supplement: Supplementary file 6 [file DataSheet1.docx]

**1. Supplementary Materials and Methods**

**1.1 Cell culture, reagents and antibodies**

Murine melanoma cells B16/F10 were obtained from the American Type Culture Collection (Manassas, VA, USA) and maintained in DMEM (Corning, 10-014-CVR) supplemented with 10% FBS (PAN, P30-3302) and 1% antibiotic. Recombinant RANTES (CCL5) was obtained from Peprotech (Rocky Hill, CT, USA). The β-actin, AKT, and p-AKT antibodies were all purchased from Cell Signaling Technology (CST, USA) and the catalogs were β-actin (8457), AKT (9272), Phospho-Akt (Ser473) (9272).

**1.2 Construction of the CCR5 knockout cell line**

The CCR5 knockout B16/F10 cell line was established using the CRISPR/Cas9 technology. The sgRNA sequence (TATAGCTATGACATCGATTATGG) was designed to target the CCR5 gene and cloned into the lentiCRISPR v2 plasmid. The recombinant plasmid was utilized for lentivirus packaging and subsequent transfection into B16/F10 cells. The transfected cells were cultured in a selective medium to facilitate the enrichment of cells with successful CCR5 knockout. To assess the knockout efficiency, CCR5 expression in the modified B16/F10 cells were evaluated using flow cytometry with the antibody CD195 (CCR5) Monoclonal Antibody (HM-CCR5 (7A4)), PE, eBioscience™ (Catalog: 12-1957-41).

**1.3 Western Blot assay**

Equal protein samples were subjected to SDS-PAGE and subsequently transferred to PVDF membranes. Following blocking with 5% nonfat milk to minimize nonspecific binding, the membranes were incubated overnight at 4 °C with primary antibodies specific to the target proteins. After washing, the membranes were incubated with HRP-conjugated secondary antibodies. Protein abundance was visualized using Clarity Western ECL Substrate (Bio-Rad) and detected with a Tanon 6100C Gel Imaging System.

**1.4 Cell treatment and preparation of cell lysates**

For CCL5-stimulated temporal phosphorylation analysis, B16/F10 cells were serum-starved with DMEM medium for 18 h when cell confluence reached 80%. Then cells were stimulated with 200 ng/mL CCL5 for different times (5, 15 and 30 min) with one group remaining no stimulation (0 min). For comparative analysis, wild type (Ctrl) and CCR5 knockout (CCR5_KO) cells were serum-starved for 18 h and then stimulated with 200 ng/mL CCL5 for 5 min with one group remaining no stimulation (0 min).

After washed with ice-cold PBS for three times, cells were lysed in buffer containing 50 mM Tris-HCl (pH 8.5), 7M Urea, 1% (v/v) Triton X-100, Protease inhibitors mixture (Complete mini, Roche) and Phosphatase inhibitor cocktail tablets (PhosSTOP, Roche). Cell lysates were processed with ultrasonic cell disruptor (SCIENTZ, JY96-IIN). After centrifugation at 14,000rpm for 10 min at 4 oC, the supernatants were collected and protein concentrations were examined through BCA protein assay kit (Pierce, 23227). For every sample, 80 μL cell lysate was set aside as an input sample for western blot analysis and 3mg cell lysates were subjected to phosphoproteomics experiment.

**1.5 Sample preparation for phosphoproteomic experiment**

Four volumes of pre-chilled methanol, one volume of pre-chilled chloroform and three volumes of pre-chilled water were sequentially added to the cell lysate, with thorough vortex after each addition. The mixture was then centrifuged at 14,000g for 2 min at 4°C. After removing the upper aqueous layer, four volumes of methanol were added, and the mixture was centrifuged again at 14,000g for 2 min to obtain the protein precipitate, which was then air-dried at room temperature for 5-10 min and redissolved in a solution of 8M urea. Then proteins were reduced with 10 mM dithiothreitol (DTT) for 25 min at 55°C and subsequently alkylated with 30 mM iodoacetamide (IAA) for 30 min at 25°C in dark, followed by the addition of 20 mM dithiothreitol (DTT) for 15 min at 25°C in dark. Samples were diluted 1:7 with 50mM Tris-HCl (pH 8.5), supplemented with 1mM CaCl2 and then digested with trypsin (Sigma, 27250018) at a 1:20 enzyme-to-substrate ratio. After digestion for 14-18 hour at 37 °C, the samples were acidified with 10% trifluoroacetic acid (TFA) to the final concentration of 0.4%TFA (pH 2-3) and centrifuged at 11,000g for 10 min at room temperature before collecting the supernatant to new tubes. Digested samples were desalted with Sep-Pak Vac tC18 1cc/50mg solid phase extraction (SPE) tubes (Waters, Milford, Massachusetts, USA) using the following protocol: 2 mL methanol was added for conditioning, followed by 1 mL of 80% acetonitrile (ACN), 0.1% TFA in H2O and 3 mL of 0.1% TFA in H2O. The samples were then loaded onto each column followed by washing with 2 mL of 0.1% TFA in H2O. Samples were eluted with 1mL of 50% ACN, 0.5% acetic acid in H2O and then dried using a SpeedVac vacuum concentrator.

Next, phosphorylated peptides were enriched using Ti4+-immobilized-metal affinity chromatography (Ti-IMAC). First, a piece of C8 membrane was placed into a 200 μL pipette tip and Ti-IMAC beads (20 Ti-IMAC: 1 peptides) were introduced into the tip by centrifuge at 2000g for 1 min. The Ti-IMAC beads were then equilibrated with 60 μL of loading buffer (80% ACN, 6% TFA) at 400g for 2 min. The lyophilized peptides were redissolved in 100mM ammonium bicarbonate (ABC), mixed with an equal volume of loading buffer, and then loaded to the Ti-IMAC beads by centrifuge at 100-200g for 10 min. After discarding the filtrate, the beads were washed twice with the washing buffer 1 (50% ACN, 6% TFA, 200mM NaCl) at 400g for 3 min each time, followed by two washes with the washing buffer 2 (30% ACN, 0.1% TFA) at 400g for 2 min each time. Phosphorylated peptides were eluted with 200 μL of 10% ammonia solution by centrifuge at 100-200g for 10-20 min and 40 μL of 50% ACN was added to elute the peptides binding to the C8 membrane by centrifuge at 800g for 3 min. The eluted phosphorylated peptides were lyophilized and desalted using C18 solid-phase extraction. After redissolved in a solution containing 4% formic acid (FA) and 5% ACN, the phosphorylated peptides were subjected to mass spectrum analysis.

**1.6 Quantitative phosphoproteomics analysis**

For CCL5-stimulated temporal phosphorylation analysis, the phosphorylated peptides were analyzed with Q Exactive HF-X mass spectrometer equipped with Easy-nanoLC (Thermo Fisher Scientific). A home-made capillary tip column (100 μm i.d. × 20 cm) was packed with ~0.5 cm of C4 (3 μm / 120 Å, Dr. Maisch GmbH) and ~20 cm of C18 resin (1.9 μm / 120 Å, Dr. Maisch GmbH). The mobile phase used for peptide separation was 0.1% (v/v) FA (solvent A) and 0.1% (v/v) FA in ACN (solvent B). The flow rate for separation was set as 250 nL/min. LC gradient for solvent B was programmed as follows: 0 min, 4%; 2 min, 8%; 57 min, 28%; 62 min, 40%; 64 min, 97%; 80 min, 97%. For intensity analysis of phosphopeptides, the mass spectrometer was operated in Top 50 DDA mode. Orbitrap mass analyzer was used to collect MS spectra with a resolution of 120,000. The range was set as 350 - 1,550 m/z; the automatic gain control (AGC) target was set as 3E6 and the maximum ion injection time (IT) was 20 ms. The most intense ions from the full scan were isolated with an isolation width of 1.4 m/z. Higher energy collisional dissociation (HCD) was performed using a normalized collision energy (NCE) setting of 25. Subsequently, MS/MS spectra were acquired in the Orbitrap mass analyzer at a resolution of 7,500, with an automatic gain control (AGC) target value set to 1E5 and a maximum ion injection time (IT) of 50 ms. Precursor dynamic exclusion was enabled with a duration of 30 s.

For comparative analysis induced by CCR5 knockout, the tryptic peptides were dissolved in solvent A, directly loaded onto a home-made reversed-phase analytical column (25-cm length, 100 μm i.d.). The mobile phase consisted of solvent A (0.1% FA, 2% ACN in water) and solvent B (0.1% FA in ACN). Peptides were separated with following gradient: 0-16 min, 2%-22%B; 16-22 min, 22%-35%B; 22-26 min, 35%-90%B; 26-30 min, 90%B, and all at a constant flow rate of 450 nL/min on a NanoElute UHPLC system (Bruker Daltonics). The peptides were subjected to capillary source followed by the timsTOF Pro mass spectrometry. The electrospray voltage applied was 1.7 kV. Precursors and fragments were analyzed at the TOF detector. The timsTOF Pro was operated in data independent parallel accumulation serial fragmentation (dia-PASEF) mode. The full MS scan was set as 100-1700 (MS scan range) and 22PASEF (MS/MS mode) -MS/MS scans were acquired per cycle. The MS/MS scan range was set as 395-1395 and isolation window was set as 20 m/z.

**1.7 Data processing and statistical analysis**

Raw mass spectrometry data obtained from Thermo Fisher mass spectrometry were processed using MaxQuant (v1.5.5.1). The DIA data obtained from timsTOF Pro mass spectrometry were processed using Spectronaut (v.18) software. For phosphopeptide identification, oxidation (M), deamidation (NQ), and phosphorylation (S, T, Y) were included as variable modifications. Carbamidomethylation (C) was specified as a fixed modification. Trypsin specificity was applied, permitting up to two missed cleavages. Peptide and protein identifications were filtered at a false discovery rate (FDR) of 1%, estimated using a target-decoy approach.

Statistical analysis between different groups was realized through Perseus software (Version 1.5.5.3). Phosphosites from reverse sequences and potential contaminants were removed and phosphosites with localization probability more than 0.75 were kept for further analysis. All intensities were log2 transformed and after invalid values were randomly replaced against the total matrix, student's T-test was operated with the threshold of FDR as 0.05 and S0 as 0.5.

In order to chart dynamic change of phosphopeptide intensity, intensities were normalized and the relative abundance to the maximum value among all time points of every phosphopeptide was calculated and dynamic change was visualized in heatmaps through R packages (Version 3.6.1). Gene Ontology enrichment was performed with R package cluster Profiler v.4.2.2 and data were visualized with R package pheatmap v.1.0.12. Reactome pathways enrichment was performed using the String database.
