# Supplementary material for: Quantitative phosphoproteomic profiling of CCL5/CCR5 signaling cascade in melanoma cells
Source: Front Oncol. 2026 Jun 22;16:1852022. doi: 10.3389/fonc.2026.1852022 (PMC13333406; doi:10.3389/fonc.2026.1852022)
Supplement: Supplementary file 7 [file DataSheet2.docx]

**Supplementary Figures**


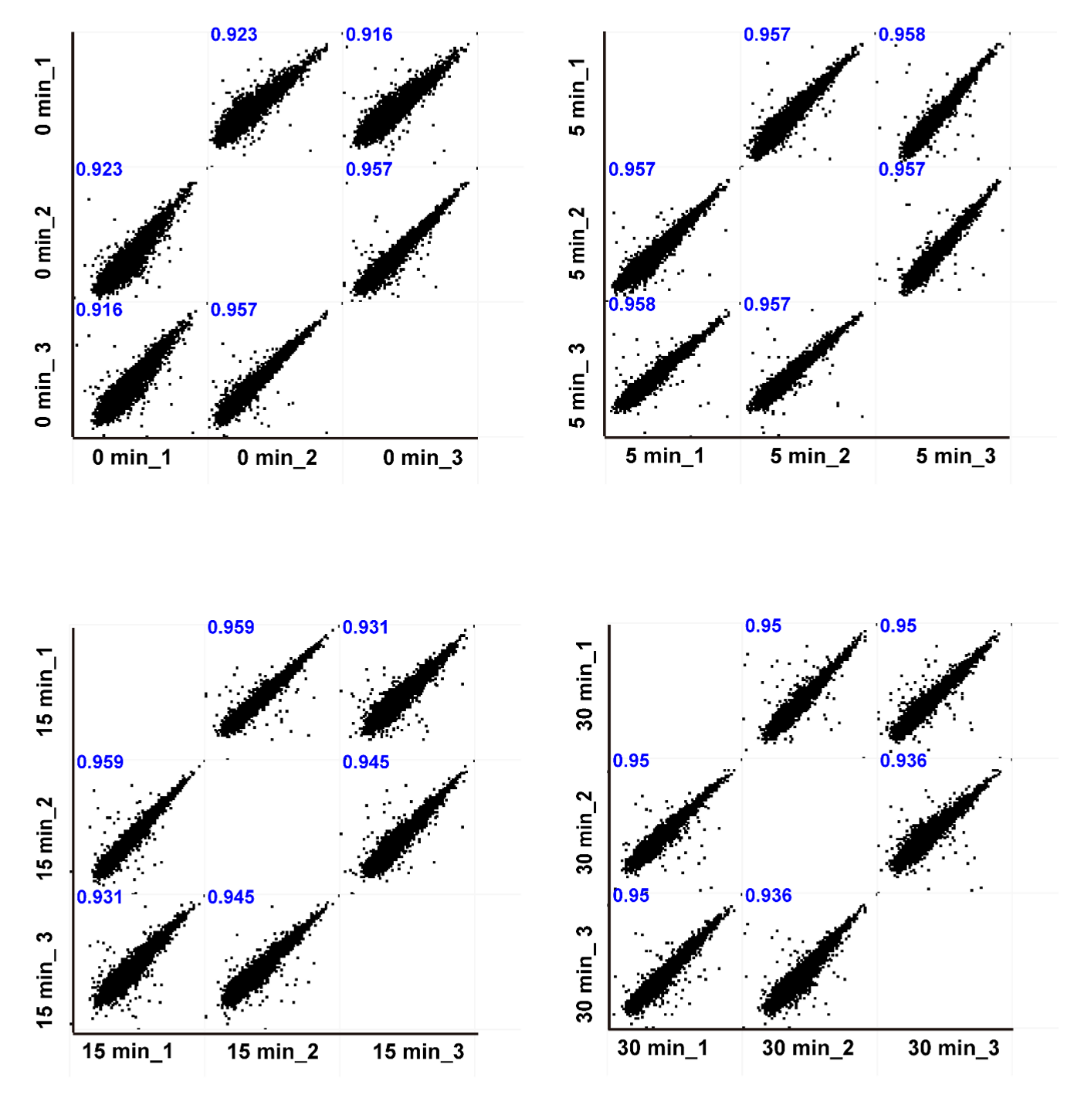


**Figure S1.** Pearson correlations between every two replicates in three biologically independent replicates.


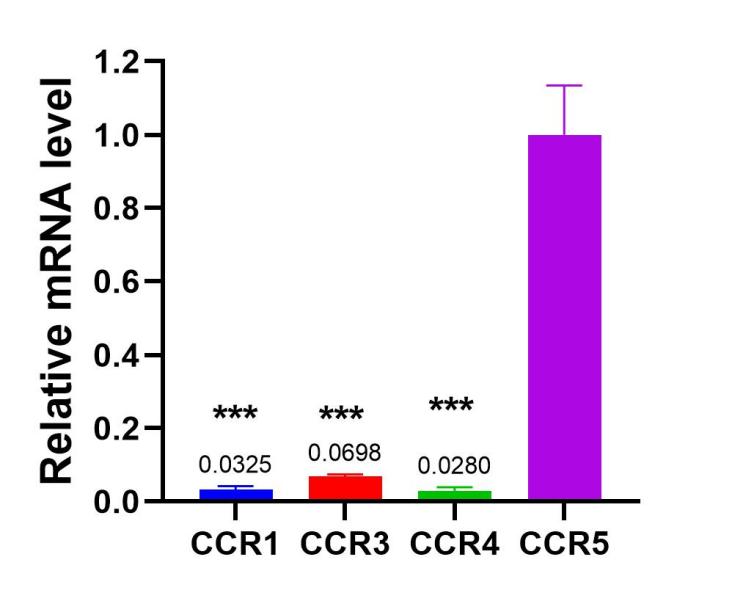


**Figure S2.** The relative mRNA expression levels of CCL5 receptors, namely CCR1, CCR3, CCR4, and CCR5, in the B16/F10 melanoma cell line were determined using quantitative real-time polymerase chain reaction (qRT-PCR). Each experiment was performed in triplicate, and statistical significance was assessed using the Student's t-test (*p < 0.05, **p < 0.01, ***p < 0.001).


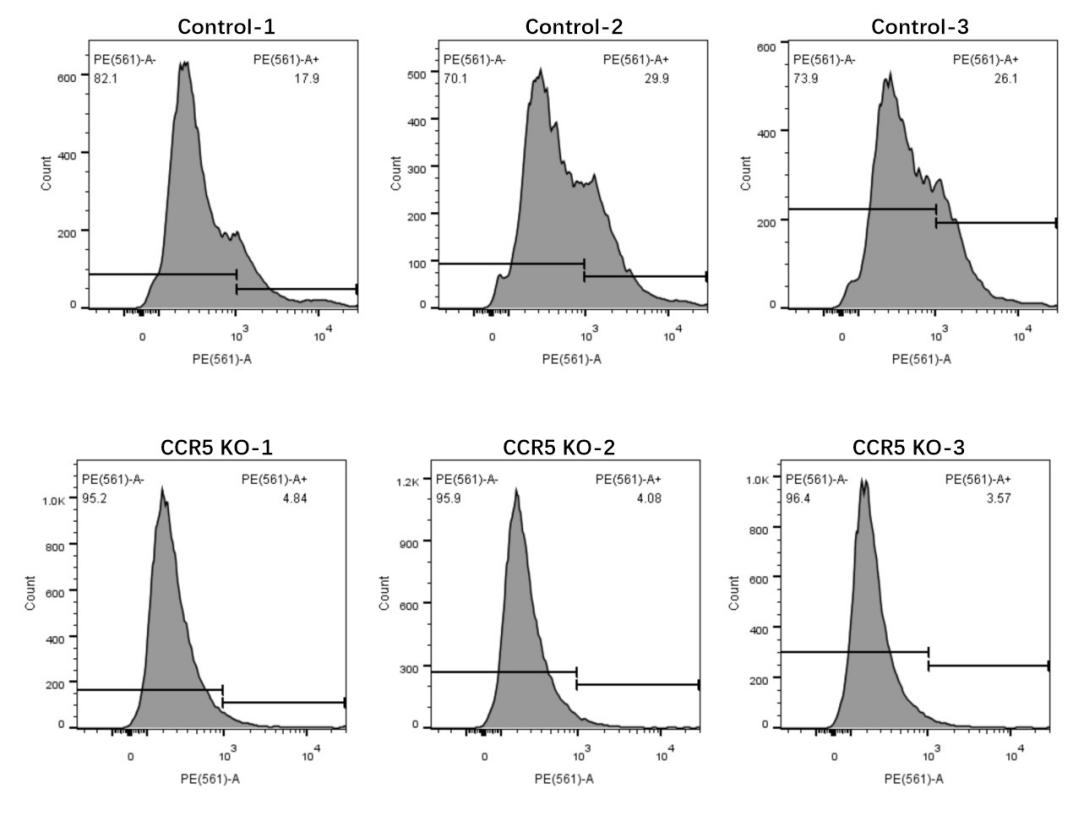


**Figure S3.** The expression levels of surface CCR5 in wild type B16/F10 cells (Control) and CCR5 knockout B16/F10 cells (CCR5 KO) were examined through flow cytometry with PE-conjugated antibody targeting CCR5 protein. The percentage of CCR5 positive and CCR5 negative cells was shown and each group was performed in triplicate.
